# Castration for Prostatic Hypertrophy

**Published:** 1899-04

**Authors:** 


Castration for Prostatic Hypertrophy. Chronic hypertrophy of the prostate gland, quite common in the senile state of man, I have also seen comparatively frequently in aged dogs.

There seems to be a developmental relationship between the testicles and the ovaries and the other genital organs with which these two are associated respectively. If the one or the other be removed the remaining genital organs suffer a certain amount of atrophy. This same phenomenon follows castration, and thus the prostate atrophies after the animal is unsexed.

Relative to this point, Drs. J. W. White and E. R. Kirby,[1] in conducting a series of experiments for the purpose of discovering a treatment of enlarged prostate in man, determined that the average weight of the prostate of the dog is $15\frac{3}{4}$ grammes; also, that if the prostate be removed from such dogs thirty to sixty days after castration, it weighs only $3\frac{9}{16}$ grammes, or only about one-fourth of its original weight. The effect of castration upon the prostate is, therefore, very decided. The same statement may be made in this respect about the other domestic species.

I know personally of only one canine patient in which castration was performed with this end in view. The patient was a Newfoundland dog, aged, and presenting the usual symptoms. Frequent attempts at urination, the urine sometimes escaping in drops or small spurts. Rectal examination with the finger revealed the prostate gland to be much enlarged and hard. The presence of vesical calculus was eliminated. The dog was castrated and discharged, and for some time has passed beyond observation. I afterward had inquiry made from the owner, and was told that the dog had made a good recovery, and the previous symptoms had disappeared.

---

The Montana State Board of Veterinary Medical Examiners will in the future require an examination of all graduates who desire to enter the State to practice.

The renowned bulldog, "His Nibs," is dead, having expired in a fit, said to have followed the bite of a copperhead snake, received while playing on the lawn of his owner.

Kansas legislation has just appropriated $34,000 for the establishment of a dairy school; $2000 for farmers' institutes, and $110,000 for the State Agricultural College.

There was a decrease of 3760 horses in the number received in the Chicago market the first two months of this year, compared with the same period in 1898. A shire gelding, weighing 2100 pounds, sold for $350 in March, showing the rising value of horseflesh.

[1] Veterinary Magazine, 1894, vol. i.

